# Supplementary material for: HIVprotI: an integrated web based platform for prediction and design of HIV proteins inhibitors
Source: J Cheminform. 2018 Mar 9;10:12. doi: 10.1186/s13321-018-0266-y (PMC5845081; doi:10.1186/s13321-018-0266-y)
Supplement: Supplementary file 2 — Additional file 2. Source code of HIVProtI web server. [file 13321_2018_266_MOESM2_ESM.zip › HIVprotI_Source-code/hivprotI/weka-3-6-6/doc/weka/core/logging/package-summary.html]

weka.core.logging


---


|  |  |  |
| --- | --- | --- |
| **Overview** \| **Package** \| Class \| **Tree** \| **Deprecated** \| **Index** \| **Help** \| **Weka's home** | |  |
| **PREV PACKAGE**   **NEXT PACKAGE** | **FRAMES**    **NO FRAMES**     **All Classes** |  |


---

## Package weka.core.logging

| **Class Summary** | |
| --- | --- |
| **ConsoleLogger** | A simple logger that outputs the logging information in the console. |
| **FileLogger** | A simple file logger, that just logs to a single file. |
| **Logger** | Abstract superclass for all loggers. |
| **OutputLogger** | A logger that logs all output on stdout and stderr to a file. |
| **OutputLogger.OutputPrintStream** | A print stream class to capture all data from stdout and stderr. |

| **Enum Summary** | |
| --- | --- |
| **Logger.Level** | The logging level. |

---


|  |  |  |
| --- | --- | --- |
| **Overview** \| **Package** \| Class \| **Tree** \| **Deprecated** \| **Index** \| **Help** \| **Weka's home** | |  |
| **PREV PACKAGE**   **NEXT PACKAGE** | **FRAMES**    **NO FRAMES**     **All Classes** |  |


---
